# Effects of livestock grazing on rangeland biodiversity: A meta‐analysis of grouse populations

**DOI:** 10.1002/ece3.3287

**Published:** 2017-08-17

**Authors:** Seth J. Dettenmaier, Terry A. Messmer, Torre J. Hovick, David K. Dahlgren

**Affiliations:** ^1^ Department of Wildland Resources Jack H. Berryman Institute Utah State University Logan UT USA; ^2^ Ecology Center Utah State University Logan UT USA; ^3^ Range Science Program School of Natural Resource Sciences North Dakota State University Fargo ND USA

**Keywords:** conservation, effect size, grassland, grouse, Hedges' g, herbivory

## Abstract

Livestock grazing affects over 60% of the world's agricultural lands and can influence rangeland ecosystem services and the quantity and quality of wildlife habitat, resulting in changes in biodiversity. Concomitantly, livestock grazing has the potential to be detrimental to some wildlife species while benefiting other rangeland organisms. Many imperiled grouse species require rangeland landscapes that exhibit diverse vegetation structure and composition to complete their life cycle. However, because of declining populations and reduced distributions, grouse are increasingly becoming a worldwide conservation concern. Grouse, as a suite of upland gamebirds, are often considered an umbrella species for other wildlife and thus used as indicators of rangeland health. With a projected increase in demand for livestock products, better information will be required to mitigate the anthropogenic effects of livestock grazing on rangeland biodiversity. To address this need, we completed a data‐driven and systematic review of the peer‐reviewed literature to determine the current knowledge of the effects of livestock grazing on grouse populations (i.e., chick production and population indices) worldwide. Our meta‐analysis revealed an overall negative effect of livestock grazing on grouse populations. Perhaps more importantly, we identified an information void regarding the effects of livestock grazing on the majority of grouse species. Additionally, the reported indirect effects of livestock grazing on grouse species were inconclusive and more reflective of differences in the experimental design of the available studies. Future studies designed to evaluate the direct and indirect effects of livestock grazing on wildlife should document (i) livestock type, (ii) timing and frequency of grazing, (iii) duration, and (iv) stocking rate. Much of this information was lacking in the available published studies we reviewed, but is essential when making comparisons between different livestock grazing management practices and their potential impacts on rangeland biodiversity.

## 1 INTRODUCTION

A recent assessment of vertebrates found one‐fifth classified as Threatened on the International Union for Conservation of Nature (IUCN) Red List (“The IUCN Red List of Species. Version 2015‐04”, 2015). On average, 52 species move one category closer to extinction each year. In 2010, most indicators of the state of biodiversity (i.e., population trends, extinction risk, habitat extent and quality, and community composition) declined, whereas the indicators of pressures on biodiversity increased (Butchart et al., 2010). Increased anthropogenic land use is implicated as a major factor in decreased biodiversity (de Baan, Alkemade, & Koellner, 2012; Jetz, Wilcove, & Dobson, 2007; Sala et al., 2000; Sisk, Launer, Switky, & Ehrlich, 1994).

Globally, livestock grazing is the predominant anthropogenic land use (Alkemade, Reid, van den Berg, de Leeuw, & Jeuken, 2013). Livestock grazing occurs on approximately 60% of the world's agricultural land and supports approximately 1.5 billion cattle and buffalo (Bovinae) and 1.9 billion sheep (*Ovis* spp.) and goats (*Capra* spp. and related species) (Alexandratos & Bruinsma, 2012). Global production of livestock for human consumption has more than doubled since the 1960s (Speedy, 2003). Concomitantly, the demand for livestock products is projected to increase 70% by 2050 in response to human population growth, increased discretionary income, and urbanization (Alexandratos & Bruinsma, 2012; Thornton, 2010).

Rangelands (i.e., grasslands, shrublands, woodlands, and tundra) are estimated to provide over 70% of the forage consumed by livestock worldwide (Lund, 2007). Rangelands also provide habitat for a diversity of wildlife species (Krausman et al., 2009). Thus, how these areas are managed can have important consequences for wildlife worldwide (Alkemade et al., 2013; Bock, Saab, Rich, & Dobkin, 1993; Jankowski et al., 2014; Kantrud & Kologiski, 1982; Krausman et al., 2009; Owens & Myres, 1973). Of particular concern, are ground nesting birds, such as grouse species (Tetraonidae), whose habitats are often associated with livestock grazing throughout the northern hemisphere. Livestock grazing has been implicated as both a source of mortality and an indirect driver of declines in habitat and populations in rangeland environments (Baines, 1996; Boyd, Beck, & Tanaka, 2014; Calladine, Baines, & Warren, 2002; Jenkins & Watson, 2001; Warren & Baines, 2004). Additionally, many of these grouse species depend on disturbances such as grazing or grazing in combination with fire during some or all of their life history, underscoring the importance of informed grazing practices (Hovick, Elmore, Fuhlendorf, & Dahlgren, 2015; McNew, Winder, Pitman, & Sandercock, 2015).

There are 20 species in the *Tetraonidae* family worldwide (Storch, 2007, 2015), 13 of which have been red listed by the IUCN (Table 1). In addition, populations for 18 of these species are declining (Storch, 2007, 2015). Habitat loss and degradation have been identified as the primary threat to grouse (Storch, 2007, 2015) and intense livestock grazing has been implicated as a conservation threat for six of the seven grouse species that occupy rangeland habitats (“The IUCN Red List of Species. Version 2015‐04”, 2015).

**Table 1:** Twenty recognized grouse species, their population estimate, population status, and population trend

Common name	Scientific name	Pop. estimate^a^	Status^b^	Trend^b^
Black Grouse^c^	*Lyrurus tetrix*	27,500,000	Least concern	Decreasing
Black‐billed Capercaillie	*Tetrao urogalloides*	<550,000	Least concern	Decreasing
Western Capercaillie	*Tetrao urogallus*	7,500,000	Least concern	Decreasing
Caucasian Black Grouse	*Lyrurus mlokosiewiczi*	<46,600	Near threatened	Decreasing
Chinese Grouse	*Bonasa sewerzowi*	Not quantified	Near threatened	Decreasing
Hazel Grouse	*Bonasa bonasia*	27,500,000	Least concern	Decreasing
Ruffed Grouse	*Bonasa umbellus*	Not quantified	Least concern	Decreasing
Dusky Grouse	*Dendragapus obscurus*	3,000,000	Least concern	Decreasing
Sooty Grouse	*Dendragapus fuliginosus*	Not quantified	Least concern	Decreasing
Greater Prairie‐Chicken^c^	*Tympanuchus cupido*	<700,000	Vulnerable	Decreasing
Lesser Prairie‐Chicken^c^	*Tympanuchus pallidicinctus*	30,000	Vulnerable	Decreasing
Sharp‐tailed Grouse^c^	*Tympanuchus phasianellus*	Not quantified	Least concern	Decreasing
Greater Sage‐Grouse^c^	*Centrocercus urophasianus*	<150,000	Near threatened	Decreasing
Gunnison Sage‐Grouse^c^	*Centrocercus minimus*	<2,500	Endangered	Decreasing
White‐tailed Ptarmigan	*Lagopus leucura*	Not quantified	Least concern	Decreasing
Willow Ptarmigan^c^	*Lagopus lagopus*	>40,000,000	Least concern	Decreasing
Rock Ptarmigan	*Lagopus muta*	>8,000,000	Least concern	Decreasing
Siberian Grouse	*Falcipennis falcipennis*	Not quantified	Near threatened	Decreasing
Spruce Grouse	*Falcipennis canadensis*	Not quantified	Least concern	Stable
Franklin's Grouse	*Falcipennis franklinii*	Not quantified	Least concern	Stable

a We report the mid‐point of population estimates.

b All status, trend, and population estimates were gathered from BirdLife International 2016.

c Species that inhabit rangelands.

As an example, the prairie grouse species that inhabit rangelands of North America are considered some of the most imperiled and at the greatest risk to improper livestock grazing practices (Silvy & Hagen, 2004). The Gunnison sage‐grouse (*Centrocercus minimus*) in North America (NA) was listed as a threatened species by the US Fish and Wildlife Service (USFWS) under the Endangered Species Act (ESA) and Endangered by the IUCN because of low population sizes, restricted range, and ongoing population decline (“The IUCN Red List of Species. Version 2015‐04”, 2015; U.S. Fish and Wildlife Service 2014). Similarly, greater and lesser prairie‐chickens (*Tympanuchus cupido* and *T. pallidicinctus*, respectively) are listed as Vulnerable. The sharp‐tailed grouse (*T. phasianellus*), once considered to have the most extensive range in NA, has declined markedly (Connelly, Gratson, & Reese, 1998; Johnsgard, 1983). Moreover, the greater sage‐grouse (*C. urophasianus*; hereafter sage‐grouse) which is listed by the IUCN as near threatened (Storch, 2015) was also considered by the USFWS for ESA protection (U.S. Fish and Wildlife Service 2015). Grazing by livestock is the predominant land use within the current sage‐grouse range and a paucity of information exists on the direct effects of grazing on these populations (Beck & Mitchell, 2000; Knick et al., 2011).

Given the projected global increase in demand for livestock production (Thornton, 2010), better information will be needed to mitigate the potential for increased impacts on rangeland ecosystems and associated wildlife species. However, our collective understanding of how grazing influences grouse species, which are often considered indicators for their ecosystems, is poorly understood despite the volumes of research that has been published about the ecology of these species (Haukos & Boal, 2016; Knick & Connelly, 2011). Therefore, a data‐driven and systematic review of the influence of grazing on grouse populations across the northern hemisphere is warranted to inform future conservation actions for these highly imperiled species.

We completed a data‐driven and systematic review of the peer‐reviewed literature to determine the current knowledge of the effect of livestock grazing on grouse populations (i.e., population indices represented by adult counts and chick production) worldwide. We used meta‐analytical methods to calculate unbiased estimates of *Hedges' g* (Hedges, 1981) as a measure of the direct effect of livestock grazing on grouse populations in addition to a categorical model meta‐analytic technique to quantify overall effects. We highlight knowledge gaps and research needs related to the effects of livestock grazing, the broadest anthropogenic land use on rangelands, on grouse populations.

## 2 MATERIALS AND METHODS

We conducted a literature search in May 2017 using the ISI Web of Science and Scopus databases. Searches were limited to peer‐reviewed journals or edited book series (e.g., Studies in Avian Biology). We developed keyword combinations to identify papers that included livestock, grazing, and grouse (Table 2). We used all terms for both title and topic searches to ensure returning the greatest number of papers possible. Common names of grouse species were included to capture studies that examined other grouse species absent from searches using the generic term “grouse.” As part of our search strategy, we included literature cited from the papers used in our analysis. No temporal or language restrictions were applied to our searches.

**Table 2:** Search terms and resulting number of publications using the ISI Web of Science and Scopus databases to locate peer‐reviewed literature assessing the effects of livestock grazing on grouse populations

	Search results (number of publications)	
Search term(s)	ISI web of science	Scopus
grouse*	3,083	2,554
(grouse* and livestock*)	64	49
(grouse* and grazing*)	107	98
(grouse* and habitat* and grazing*)	76	65
(prairie‐chicken* and livestock*)	8	9
(prairie‐chicken* and grazing*)	23	21
(prairie‐chicken* and habitat* and grazing*)	20	17
(capercaillie* and livestock*)	5	3
(capercaillie* and grazing*)	8	3
(capercaillie* and habitat* and grazing*)	6	1
(ptarmigan* and livestock*)	3	3
(ptarmigan* and grazing*)	6	8
(ptarmigan* and habitat* and grazing*)	4	5

In cases of irregular plurals, “*” allows search engines to retrieve all forms of the root word.

### 2.1 Study inclusion criteria

**Figure 1:**
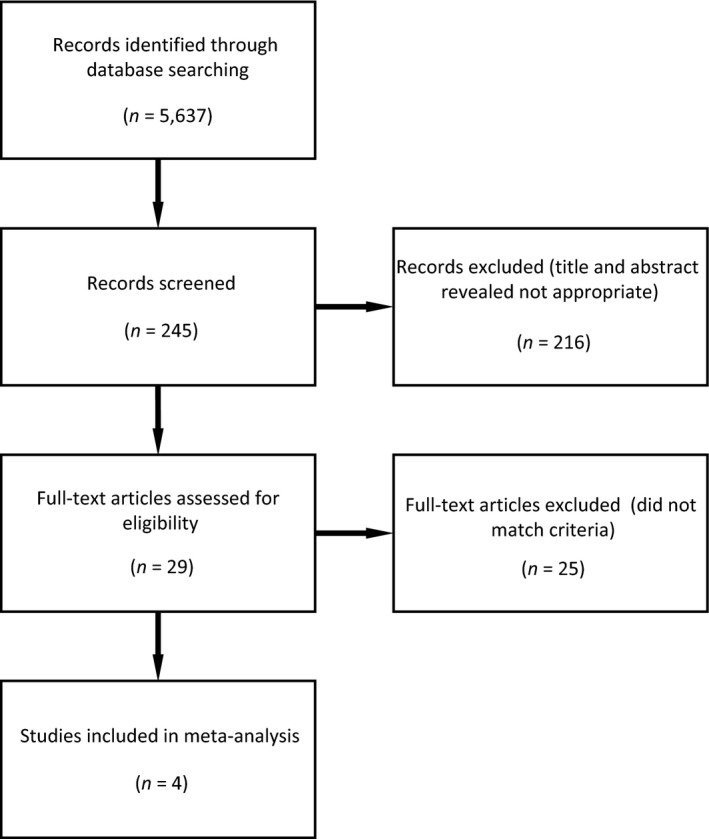
Preferred Reporting Items for Systematic reviews and Meta‐analyses (PRISMA) diagram illustrating study selection process

To refine our search, we removed papers that lacked our specific search terms within the title, abstract, or keywords. We then reviewed the remaining papers to determine whether they quantified and reported the effects of livestock grazing on grouse populations. Finally, we only retained papers that compared grouse population metrics within ≥2 grazing intensities (e.g., heavy grazing, reduced grazing, or no grazing) for the meta‐analysis. Of the initial 5,637 topic search results, only four studies met our inclusion criteria (Figure 1).

### 2.2 Data extraction

Because of the limited number of published papers that met our search criteria, we maximized the number of metrics obtained from each study. For example, Baines (1996) and Calladine et al. (2002) each reported grazing effects on both adult counts (a population indices comprised of the total males counted on leks) and chick production (chicks per female). In each study, direct effects were independently determined and analyzed separately in the meta‐analysis. Finally, one study (Jenkins & Watson, 2001) involved two species of grouse and were separated in the analysis.

### 2.3 Meta‐analysis

We quantified the direct effects of livestock grazing on grouse populations using calculated effect sizes with analyses similar to Hovick, Elmore, Dahlgren, Fuhlendorf, and Engle (2014). We standardized the reported results from each study by estimating effect sizes using the means, standard deviation, and sample sizes. To control for small sample size bias, we used *Hedges' g* effect sizes (Hedges, 1981) calculated using “compute.es” package (Del Re, 2013) in the R 3.2.3 programming environment (R Development Core Team 2015). Because field studies often lack true treatment and control levels (Hovick et al., 2014) and quantifiable grazing intensities, we categorized groups of grouse from each study into either higher‐intensity grazing sites or reduced or absent grazing sites. All meta‐analytic models were calculated using MetaWin 2.1.5 (Rosenberg, Adams, & Gurevitch, 2000). Generally, effect sizes are interpreted as <|0.2| low, |0.5| moderate, and >|0.8| high (Cohen, 1988).

Because our meta‐analysis relied on small sample sizes, we ran bootstrapping replications with replacement to improve approximations of the confidence intervals (Efron & Tibshirani, 1986). We analyzed these data using a categorical random‐effects model in Meta‐Win 2.1.5. We selected a categorical model based on the separation of our data into two distinct population measurement groups, adult counts (population indices) and chick production. Because studies differed spatially, temporally, by grazing system, and level of grazing pressure, there may be different effect sizes underlying each (Borenstein, Hedges, Higgins, & Rothstein, 2010). To address variation in the true effect size of livestock grazing based on the unique environmental and temporal factors of each study, we selected a random‐effects model. Weighted averages were used in the models to estimate the cumulative effect size by calculating the reciprocal of each studies' sampling variance, *w*_*i*_ = 1/*v*_*i*_. Because individual studies within a meta‐analysis often vary in sample size, weighting becomes necessary (Rosenberg et al., 2000). We calculated the percentage of total variation across studies that is due to heterogeneity using the *I* ^2^ statistic (Borenstein, Hedges, Higgins, & Rothstein, 2009).

We tested for publication bias, or the “file drawer problem” (i.e., when only studies reporting significant results are published) using the approaches developed by Egger, Smith, Schneider, and Minder (1997). Egger's test uses linear regression in which the standardized effect estimate *z*_*i*_ is regressed against its precision prec_*i*_ (Rothstein, Sutton, & Borenstein, 2006):

$$E\left[z_{i}\right]=\beta_{0}+\beta_{1}{\text{prec}}_{i}$$

## 3 RESULTS

**Figure 2:**
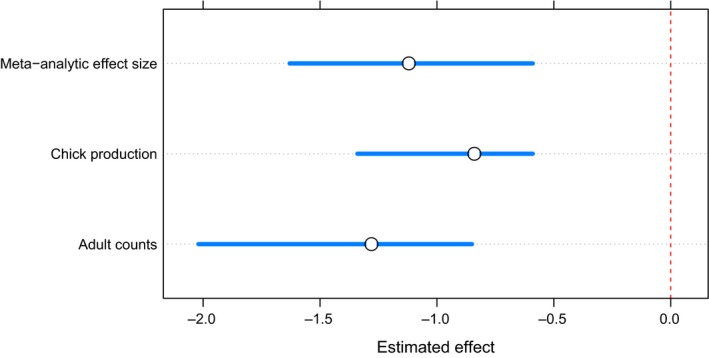
Livestock grazing had a negative effect on *Lagopus lagopus scotic*a and *Lyrurus tetrix* adult counts and chick production. Estimated effect sizes (circle) and 95% confidence interval (line) of mixed‐effects model results for adult counts, chick production, and pooled mean effect size

We analyzed six measurements of grazing's effect on adult grouse numbers and three on chick production. Our results demonstrated that livestock grazing had a negative impact on adult grouse numbers (random effects $\overset{¯}{E}$ = −1.28, *df* = 5, 95% CI: −2.02, −0.85). Additionally, we estimated a negative effect of livestock grazing on grouse chick production (random effects $\overset{¯}{E}$ = −0.84, *df* = 2, 95% CI: −1.34, −0.59). Based on these studies, there is evidence supporting an overall moderate to high negative effect of livestock grazing on adult grouse numbers and chick production (random effects $\bar{\bar{E}}$ = −1.12, *df* = 8, 95% CI: −1.63, −0.59) (Figure 2).

**Figure 3:**
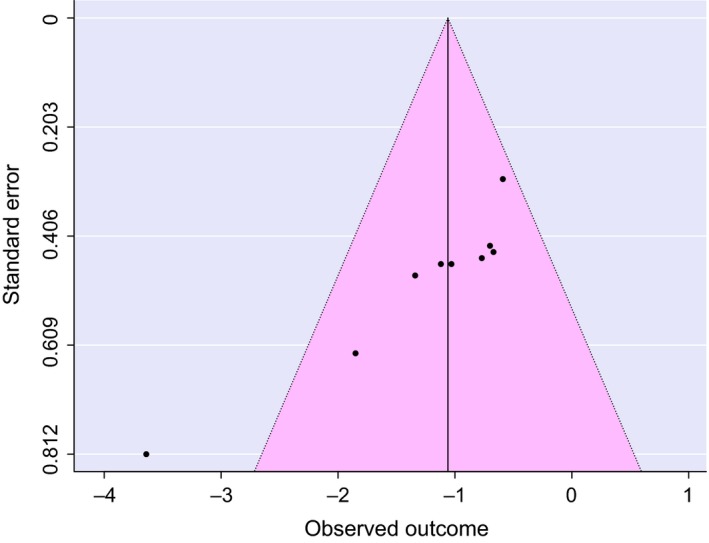
Studies meeting selection criteria demonstrate potential publication bias. Funnel plot of reported effect sizes against precision illustrates the asymmetry and potential bias of study results

We tested total proportion of variance owing to heterogeneity (*I* ^2^ = 12.5%, *df* = 8) for both adult counts and chick production. Our results indicate that the variance among effect sizes were within expected sampling error (Cooper, 1998) and that grazing level is a valid explanatory variable for the model. However, results of Egger's test (*z* = −3.62, *p* = .0003) showed that publication bias was an issue within our meta‐analysis (Figure 3).

## 4 DISCUSSION

Rangelands provide habitat for a diversity of wildlife and grouse species (Krausman et al., 2009). Livestock grazing is not only the predominant use of rangelands (Alkemade et al., 2013), but has been implicated in declines of grouse populations (Baines, 1996; Boyd et al., 2014; Calladine et al., 2002; Jenkins & Watson, 2001; Warren & Baines, 2004). Our investigation of the influence of grazing on grouse found an overall negative effect on both adult counts and chick production for two populations of European grouse species that are in decline (Baines, 1996; Calladine et al., 2002; Jenkins & Watson, 2001; Jouglet, Ellison, & Léonard, 1999; Storch, 2015). The largest reported individual effect was on adult numbers that resulted from the introduction of heavy sheep grazing into a previously ungrazed area which negatively altered the native vegetation composition (Jenkins & Watson, 2001). This review of the effects of grazing on wildlife suggests that grazing has a general negative effect on the studied grouse populations, and presents some concern for grazing in areas where grouse conservation is a main objective. However, the number of studies that reported a measurable effect of grazing on adult counts and production was limited and many considerations of grazing management warrant discussion.

**Figure 4:**
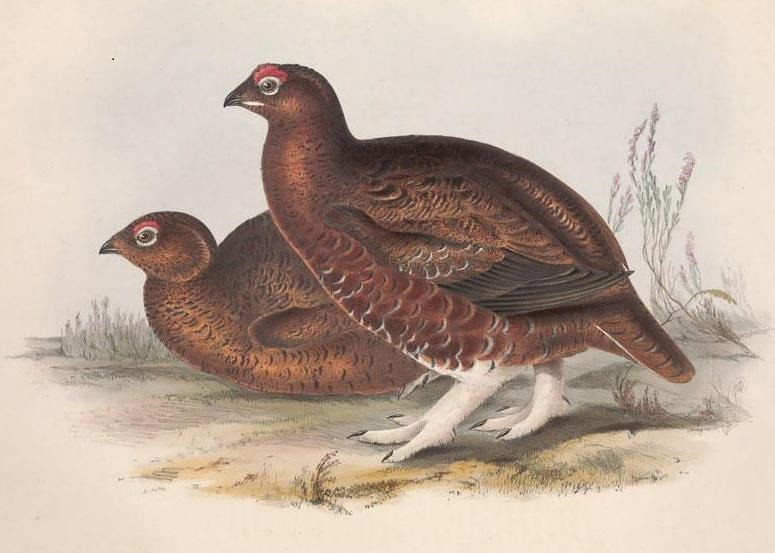
Often considered a subspecies of the willow grouse (*Lagopus l. lagopus*), red grouse (*Lagopus lagopus scotica*) are endemic to the heather moorlands of Great Britain

These studies lend support to concerns that livestock grazing management focused on maximizing meat production through high stocking rates can negatively impact grouse populations (Beck & Mitchell, 2000; Boyd et al., 2011; Silvy & Hagen, 2004) and other wildlife species (Krausman et al., 2009). Our analysis was limited to studies of black (*Lyrurus tetrix*) and red (*Lagopus lagopus scotica*) grouse (Figure 4) and lacked studies for NA prairie grouse, Arctic species of ptarmigan, and the forest species of Eurasia. Also, the total number of papers meeting our criterion were limited. There was much specific information on grouse ecology that was lacking from our dataset. This paucity of information highlights a need for more research that directly measures the effects of livestock grazing on grouse. Also, despite efforts to limit issues of publication bias within our meta‐analysis, we could not overcome the scarcity of appropriate studies in the published literature.

There was consensus in the published literature that overgrazing of rangelands by livestock has predominately negative effects on wildlife and their habitats (Boyd et al., 2011; Krausman et al., 2009; Silvy & Hagen, 2004). However, our meta‐analysis highlighted the general lack of knowledge of the direct effects of livestock grazing needed to develop best management practices (BMPs) for grouse in general and individual species specifically. With so few published studies, it is inappropriate to make broad general statements regarding the impact of livestock grazing on grouse and the BMPs for the conservation of rangelands and grouse populations without further research (Boyd et al., 2011).

The studies we analyzed were missing specific information regarding grazing management practices. They also lacked consistency in the reporting of quantifiable stocking rates for both the treatment and control groups (Baines, 1996; Jenkins & Watson, 2001). Although Calladine et al. (2002) and Jouglet et al. (1999) provided stocking rates for both the treatment and reference sites, this information was not included in their analysis. Additionally, stocking rates were not comparable across biomes. Understanding the effects of stocking rates in similar vegetation communities can help inform land‐use management decisions regarding the effect of grazing management on wildlife (Dahlgren et al., 2015; Krausman et al., 2009).

Livestock grazing systems are a complex combination of factors that include animal type, stocking rate, animal distribution, timing, duration, frequency, and many more (Briske et al., 2008; Heitschmidt & Walker, 1996; Teague et al., 2008; Veblen, Nehring, McGlone, & Ritchie, 2015; Veblen & Young, 2010). Livestock grazing may not be invariably “good” or “bad” for wildlife—rather, there can be positive, negative, or benign effects dependent on aforementioned factors in combination with soil conditions, precipitation, plant community, and the organism of concern (Krausman et al., 2009). Livestock grazing can have direct negative effects on grouse including destruction of habitat, trampling eggs, nest abandonment, and reducing food availability (Beck & Mitchell, 2000). While direct effects are often infrequent (Hovick et al., 2012), indirect effects can be more common and include conversion of habitat to forage, introduction of invasive plant species (Beck & Mitchell, 2000), and subsidizing increased predator densities (Coates et al., 2016).

The role of human dimensions in grazing systems can indirectly contribute to the ecological outcome of grazing systems (Briske et al., 2011). The manner in which livestock grazing is managed affects the structure of rangeland ecosystems, which in turn influences the flows of other ecosystem goods and services from rangelands and ultimately affects wildlife populations (Dahlgren et al., 2015; Heitschmidt & Walker, 1996; Veblen et al., 2015). While grazing has been a part of many researched systems, its effects on wildlife populations are rarely investigated in an explicit and rigorous scientific manner. The effects of livestock grazing are generally diffuse across large landscapes and research of these effects will need to occur on scales that encompass those vast landscapes (Knick et al., 2011).

Future research investigating the effects of livestock grazing on wildlife populations should account for the complex ecological landscape of rangelands. For future research, we provide the following recommendations. Studies should document the (i) livestock type, (ii) timing and frequency of grazing, (iii) duration, and (iv) stocking rate. For example, livestock type has been demonstrated to differentially affect plant composition (Rook et al., 2004) while timing and duration affect vegetation structure (Fischer et al., 2009; Hockett, 2002). These habitat changes have been demonstrated to ultimately affect wildlife biodiversity on rangelands (Alkemade et al., 2013; Krausman et al., 2009). The implementation of standardized measures of vegetation composition cover and height across all studies would help in quantifying the effects on wildlife habitats. Additionally, researchers may need to account and control for other drivers of population and habitat change such as climate and predators (Fuhlendorf, Briske, & Smeins, 2001; Guttery et al., 2013).

## CONFLICT OF INTEREST

None declared.

## AUTHORS' CONTRIBUTIONS

Seth Dettenmaier, Terry Messmer, Torre Hovick, and Dave Dahlgren conceived the ideas and designed the methodology; Seth Dettenmaier collected and analyzed the data; and Seth Dettenmaier, Terry Messmer, Torre Hovick, and Dave Dahlgren contributed critically to the drafting and revision of the manuscript and gave final approval for publication.

## DATA ACCESSIBILITY

All data used in this study were sourced from published studies.
